# Escape or Fight: Inhibitors in Hemophilia A

**DOI:** 10.3389/fimmu.2020.00476

**Published:** 2020-03-24

**Authors:** Simone Merlin, Antonia Follenzi

**Affiliations:** ^1^Laboratory of Histology, Department of Health Sciences, Università degli Studi del Piemonte Orientale “A. Avogadro”, Novara, Italy; ^2^Interdisciplinary Research Center of Autoimmune Diseases (IRCAD), Novara, Italy

**Keywords:** inhibitor, immune tolerance induction, factor VIII, hemophilia A, immune modulation, regulatory T cells

## Abstract

Replacement therapy with coagulation factor VIII (FVIII) represents the current clinical treatment for patients affected by hemophilia A (HA). This treatment while effective is, however, hampered by the formation of antibodies which inhibit the activity of infused FVIII in up to 30% of treated patients. Immune tolerance induction (ITI) protocols, which envisage frequent infusions of high doses of FVIII to confront this side effect, dramatically increase the already high costs associated to a patient's therapy and are not always effective in all treated patients. Therefore, there are clear unmet needs that must be addressed in order to improve the outcome of these treatments for HA patients. Taking advantage of preclinical mouse models of hemophilia, several strategies have been proposed in recent years to prevent inhibitor formation and eradicate the pre-existing immunity to FVIII inhibitor positive patients. Herein, we will review some of the most promising strategies developed to avoid and eradicate inhibitors, including the use of immunomodulatory drugs or molecules, oral or transplacental delivery as well as cell and gene therapy approaches. The goal is to improve and potentiate the current ITI protocols and eventually make them obsolete.

## Introduction

The major complication of replacement therapies in hemophilia A (HA) is the formation of inhibitors, anti-FVIII antibodies directed against and inhibiting the function of infused FVIII. The formation of inhibitors occurs in ~30% of HA patients as a severe form, and in ~5% of patients as mild/moderate forms (1, 2). Should inhibitor formation occur, it will do so within 75 exposure days in patients with severe HA (3).

To date the only clinical option for inhibitor eradication is immune tolerance induction (ITI) protocols, which consist of frequent infusions of FVIII. According to the current protocols, high doses of FVIII are administered daily (Bonn protocol: 100–150 IU/kg FVIII twice a day) (4) or every other day (Creveld protocol: 25 IU/kg FVIII every 2 days) (5). Depending on the patient's response, the period of treatment will vary from months to over 1 year, with a successful outcome seen in ~70% of treated patients (6). Despite the high success rate and safety reported, the long treatment period using a central venous catheter for frequent infusions, as well as the high costs, are the major drawbacks of this treatment.

**Figure 1:**
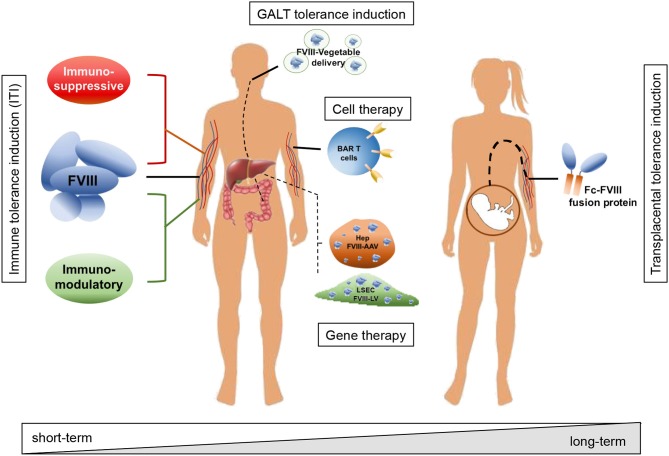
Schematic representation of strategies adopted to avoid inhibitor formation and to induce tolerance toward FVIII. Strategies include immune modulatory drugs and molecules acting on T and B cells (e.g., rapamycin, dexamethasone, anti-CD20, IL-2/IL-2mAb complexes); interaction with the GALT and tolerance induction through oral administration of FVIII peptides bioencapsulated in plant cells; tolerization at fetal stage through transplacental delivery of FVIII to the pregnant mother; adoptive transfer of FVIII-sensitized Tregs and/or expression specific chimeric antigen receptors (CAR) and engineered B-cell antibody receptors (BAR) expression on T cells; targeted gene therapy for FVIII expression in organs or cell types able to modulate immune reactions and induce tolerance to the transgene, e.g., hepatocytes and liver sinusoidal endothelial cells (LSEC). According to the adopted strategy, the treatment can result in a short-term effect, requiring more administrations and time to achieve tolerance, or in a long-term effect, with virtually life-long tolerance to FVIII with a single administration.

The recent introduction of emicizumab, a bispecific antibody directed against FIXa and FX which mimics the FVIII function, has offered a new approach to the management of ITI. This approach allows the use of lower doses of FVIII and reduces the frequency of administration (7, 8). There remains, however, a need for effective options to treat ITI refractory patients. As such, novel strategies to prevent or eradicate inhibitor formation are required. Different approaches have been proposed in recent years aimed at avoiding the formation of or eradicating existing inhibitors, including the use of immunomodulatory drugs or molecules, oral or transplacental delivery as well as cell and gene therapy approaches, taking advantage of preclinical models of HA (Figure 1).

## Tolerance Induction by Immune Suppression

A possible approach to induce tolerance toward FVIII is guiding the immune system toward a FVIII-specific regulatory T cell (Treg) response, thus suppressing T and B cells reacting against FVIII. Some of these approaches are represented by immunomodulatory drugs or molecules that favor the activation of Treg and inhibit the activation of effector T cells *in vivo*.

Rapamycin, also known as sirolimus, is an antibiotic able to inhibit the mammalian target of rapamycin (mTOR), which reduces cell cycle progression and suppresses effector T cell proliferation, thus rendering this molecule a useful immunosuppressor for allograft transplantation. Moreover, administration of rapamycin results in Tregs expansion, depending on the treatment time and dosage (9–12). In a previous study by Moghimi and colleagues, the daily oral administration of rapamycin for 1 month with the concurrent administration of FVIII, both B-domain deleted-FVIII (BDD-FVIII) or full length FVIII, was able in HA mice to prevent inhibitor formation following weekly FVIII infusions over a 3.5 months period. In control HA mice lacking the administration of rapamycin, the same treatment with FVIII resulted in a high titer inhibitor formation. In this case, a tolerization protocol stimulated Tregs which were able to, upon adoptive transfer from treated mice in naïve HA mice, avoid inhibitor development following immunization. Further, co-administration of FVIII during rapamycin treatment was found to be essential for FVIII tolerization since mice receiving only rapamycin developed high-titer inhibitors within 1 month after weekly FVIII administration following the rapamycin regimen (13).

Since differentiation of naïve CD4 T cells into regulatory or effector T cells is associated with the type and activation status of dendritic cells (DC) (14), administration of determined cytokines in association with rapamycin can further shift the balance toward Treg differentiation. For example, FMS-like receptor tyrosine kinase 3 ligand (Flt3L) administration results in DC expansion and induction of Tregs in both humans and mice (15). According to this observation, rapamycin in combination with Flt3L and low doses (0.3 IU) of FVIII and subsequent treatment for FVIII therapy (1 IU weekly) in a HA mouse model, was able to significantly reduce inhibitor formation by promoting Treg induction (16).

Suppression of pro-inflammatory signals during initial exposures to FVIII has been shown to reduce the incidence of inhibitor development in a number of studies (17–19). Transient treatment with dexamethasone, an anti-inflammatory and immunosuppressive corticosteroid, in conjunction with FVIII, was able to significantly reduce the development of anti-FVIII antibodies in HA mice as well as in a mouse model of HA with humanized major histocompatibility complex (MHC) type II transgene. Additionally, among mice negative for anti-FVIII antibodies after initial FVIII exposure, dexamethasone-treated mice were less prone to develop anti-FVIII immune response following a re-challenge at 6 and 16 weeks (20, 21). This antigen-specific tolerance induced by transient dexamethasone treatment was associated with an increase in thymic Tregs (21).

The use of short term/transient treatments of HA mice with other safe and well-tolerated immunomodulatory agents (22–24), such as anti-CD20 (25, 26), anti-CD3 (27, 28) or IL-2/IL-2mAb complexes (29), have shown significant effects in the prevention of inhibitor formation.

Other than T cells, B cells represent an additional target for FVIII tolerance induction. It was observed that B cell depletion with a single dose of IgG1 anti-CD20 in mice previously immunized with FVIII, avoided an increase of inhibitor titers for FVIII, thus resembling the high dosage protocols for ITI. IgG1 anti-CD20 treatment resulted in increased splenic Tregs and was efficacious for up to 3 months after a partial B cell depletion (26). In a more recent study in mice, the combined treatment of rapamycin and IgG2a anti-CD20 was able to reduce the inhibitors from a high titer (~10 BU/ml) to a low titer (≤ 5 BU/ml), with a minimal increase in inhibitor titers following a FVIII re-challenge after B cell repopulation (25).

In other studies which examined the administration of interleukin 2 (IL-2) bound to a particular anti-IL-2 monoclonal antibody (mAb; JES6), known as IL-2/IL-2-mAb complexes, it was observed that these complexes were able to selectively expand Tregs *in vivo* (30, 31). When administered concomitantly with low doses of FVIII, IL-2/IL-2-mAb complexes were shown to be effective in abrogating the development of anti-FVIII antibodies, as well as inducing the long term tolerance to FVIII in HA mice without affecting the immune reactivity of T cells to other antigens (29).

Overall, each of the pre-clinical studies described herein, highlight the importance of inducing tolerance to FVIII in a preventive manner and that with additional studies, these strategies have the potential to be adopted in clinical trials for the management of HA patients. Even though these treatments are able to induce tolerance to FVIII for long term, they are not able to guarantee a lifelong tolerance for the replacement therapy. Therefore, there is a need of new strategies aiming to induce a definitive tolerance to FVIII.

## Transplacental Delivery of Fc Fusion Protein

Since the highest risk of inhibitor development occurs within the first 15–20 exposure days in HA patients and there is the need to start early with FVIII infusions, Lacroix-Desmazes and colleagues proposed to induce tolerance prior to beginning the FVIII replacement therapy (32). This approach relies on maternal IgG crossing the placental barrier through a transcytosis mechanism, which is based on the binding of IgG to the neonatal Fc receptor (33). This mechanism allows the IgG passage from the maternal to the fetal circulation and occurs during the third trimester of fetal development, the period in which the fetal immune system develops and acquires tolerance to self (34–36). Being an ideal timing for tolerance induction to FVIII, Lacroix-Desmazes' group generated immunodominant FVIII domains, A2 and C2, fused to mouse Fcγ1 (A2Fc and C2Fc) and co-injected them into pregnant HA mice at 16, 17, and 18 days of gestation. Starting at 6 weeks of age, offspring treated with A2Fc and/or C2Fc with FVIII, showed lower anti-A2 and anti-C2 antibody titers (~10 fold) along with a significant reduction (7–8-fold) in inhibitor development, when compared to the control group. Moreover, they observed a significant reduction in the proliferation of splenic cells (isolated from A2+C2-tolerized mice) in the presence of FVIII. This suggests that there is an induction of FVIII-specific Tregs that are able to significantly reduce *in vitro* the proliferation of effector T cells from mice immunized with FVIII and *in vivo* the antibody response to FVIII upon adoptive transfer of CD4+CD25+ from FVIII-tolerized mice into naïve HA mice (32).

Overall, the use of the FVIII-Fc fusion protein already present in the market (37) could be a potential prenatal treatment of HA patients to induce FVIII tolerance which lasts a sufficient amount of time to reduce/avoid inhibitor formation. Issues remain, however, which must be addressed including treatment timing and dosage and in particular the ability of FVIII-Fc to bind vWF in which is a larger complex to transfer (38).

## Oral Tolerance Induction

Protocols able to induce tolerance toward FVIII in HA patients while avoiding immune suppression and/or toxicity would be ideal and would improve patient compliance. Within the body, the small intestine is exposed to a massive number of antigens of both intestinal bacteria and dietary origin. In order to avoid potentially damaging pro-inflammatory immune responses, the gut-associated immune system (GALT) favors an environment promoting tolerance, especially to food antigens (39). Taking advantage of this naturally occurring immune tolerant environment, tolerance induction toward a determined antigen, including FVIII, is possible. Previous studies from Rawle and colleagues, showed that mucosal administration of purified FVIII C2 domain (FVIII-C2) followed by immunization with FVIII-C2 or full length FVIII, significantly reduced titers of anti-FVIII-C2 antibodies in HA mice, thus obtaining a tolerance to FVIII-C2 that was transferred to naïve HA mice upon CD4+ splenocyte adoptive transfer. The effect, however, of this induced tolerance was temporary since the re-challenge with FVIII-C2 4 weeks later, resulted in inhibitor development in tolerized mice (40). The issues related to this approach for clinical use are the costs related to the antigen production and purification, as well as the requirement of protecting the antigen from degradation within the stomach following oral administration while efficiently reaching the GALT. From this point of view, the production of bioactive proteins in plants presents several advantages, such as low cost, a high scale production, maintenance of post-translational modifications (e.g., N-glycosylation) and absence of endotoxins (41). Furthermore, taking advantage of their cell walls, plant cells offer a natural encapsulation for antigens that need to be released in the intestine (38, 41). In 1999, Hooker and colleagues were able to express active full length FVIII in a transgenic tobacco line (41). While in 2014, FVIII heavy chain (HC) and C2 domains were produced in the tobacco chloroplast as a fusion protein with the subunit B of cholera toxin, a transmucosal carrier, and with the antigens encapsulated in plant cells for their protection during an oral delivery. Mice fed with plant material containing FVIII antigens and subsequently immunized with weekly FVIII intravenous injections showed significantly lower inhibitor titers (~7-fold) compared to control mice. Moreover, oral delivery of FVIII antigens was able to revert a pre-existing immunity to FVIII by significantly reducing inhibitor titers during 2–3 months of feeding, with a subsequent analysis suggesting the activation of Tregs in tolerized mice when compared to control animals (42). More recently, full length FVIII was expressed at optimal levels in lettuce chloroplasts and the oral delivery of FVIII produced in lettuce was shown to be able to significantly reduce inhibitor formation and induce Tregs (43). In both systems, tobacco and lettuce chloroplasts, exogenous proteins were produced at high levels and were correctly folded, although N-glycosylation was absent. Despite this, FVIII production and bioencapsulation in different plant systems offers advantages, including a reduction in costs associated to cell culture systems and the possibility of a long-term storage of plant cells (freeze-dried) for oral delivery without affecting the structure or the activity of the exogenously produced protein (43).

## T Cell Therapy

Even though the cell mechanisms leading to inhibitor development are not completely clear, it has been identified that it is a mechanism involving T helper cells (44, 45), with Tregs playing a pivotal role in tolerance to FVIII replacement therapy (46, 47). As described above, simultaneous administration of FVIII and immunomodulatory drugs/molecules results in the deletion of T effector cells (Teff) and the induction and/or expansion of Tregs (26–29). For these reasons a possible strategy for FVIII tolerance induction may consider the use of FVIII-specific Tregs.

There are two main distinct subsets of Tregs: naturally occurring, or thymic, Tregs (nTregs), which are specific mainly for self-antigens, and peripherally induced Tregs (iTregs), presenting specificity for exogenous antigens (46, 48). While the use of nTregs is restricted by the antigen non-specificity and the low recurrence, the use of iTregs represents a more realistic strategy to achieve tolerance to FVIII (46). A previous study using HA mice showed that the adoptive transfer of autologous polyclonal Tregs expanded *ex vivo* was able to strongly decrease and even suppress inhibitor development in a dose-dependent manner (49). On the contrary, the use of FVIII-specific Tregs is more efficient at lower frequencies. Recently, Smith and colleagues showed that FVIII-specific Tregs, isolated from FVIII-sensitized mice and expanded *in vitro* in presence of FVIII, have a superior ability in suppressing anti-FVIII immune response in FVIII plasmid-treated HA mice, even following a second treatment with FVIII plasmid, and promoting long term tolerance to FVIII (50). As an alternative approach, Herzog and colleagues isolated CD4+ T cells from FVIII immunized HA mice, engineered them with a retroviral vector for the expression of Foxp3 and finally adoptively transferred into naïve HA mice followed by weekly injections of FVIII for 2 months. Foxp3-transduced cells from FVIII immunized mice (Foxp3^FVIII^) were able to induce tolerance during the FVIII infusion time, avoiding inhibitor formation. Even though this approach was not able to revert pre-existing immunity to FVIII, the combination of Foxp3^FVIII^ adoptive transfer and treatment with anti-mCD20 was able to reduce pre-existing inhibitor titers (51).

These studies highlight the need of FVIII-specific cells in order to reach a more reliable and long lasting FVIII tolerance. From this point of view a finer tuning can be achieved taking advantage of specific chimeric antigen receptors (CAR) and engineered B-cell antibody receptors (BAR) expression on T cells. In a recent study, Yoon and colleagues described the generation of an engineered FVIII A2 domain-specific CAR (ASN8 CAR) and transduction of Tregs with ASN8 CAR sequence using a retroviral vector. ASN8 CAR-transduced Tregs were able to proliferate in the presence of FVIII and suppress the proliferation of FVIII-specific T effector cells *in vitro*. When injected in mice immediately after immunization with FVIII, *in vitro* expanded ASN8 CAR-transduced Tregs were able to effectively suppress anti-FVIII antibody development for up to 8 weeks, even though transplanted cells were already undetectable after 2 weeks. However, 8 weeks after adoptive transfer a re-challenge with FVIII resulted in anti-FVIII antibody development, thus meaning a loss of tolerance (52). More recently, the same group generated cytotoxic T cells expressing a CAR containing the immunodominant A2 and C2 domains of FVIII able to target FVIII-specific B cells (BAR T cells). A2 and C2 BAR T cells alone showed the ability to partially reduce anti-FVIII antibodies secreting cells *in vitro*, while when used in combination A2/C2 BAR T cells reduced anti-FVIII antibodies secreting cells almost completely. *In vivo* administration of A2/C2 BAR T cells in HA mice followed by immunization with FVIII resulted in prevention of anti-FVIII antibody formation even after a re-challenge with FVIII 10 weeks later. Moreover, analysis of splenocytes from mice 12 weeks after injection of A2/C2 BAR T cells showed the absence of FVIII-specific memory B cells, confirming that A2/C2 BAR T cells were able to prevent anti-FVIII antibody formation probably by eliminating FVIII-specific memory B cell precursors (53).

This data suggests that adoptive transfer of FVIII-specific Tregs, possibly in combination with specific chimeric antigen receptors leads to FVIII tolerance. This data is encouraging and offers a feasible approach for the prevention/management of inhibitors in HA patients.

## Gene Therapy

Hemophilia A is an X-linked bleeding disease caused by reduced or absent activity of coagulation factor (F) VIII which is a consequence of mutations or deletions within the F8 gene. Since it is a monogenic disease, HA represents an ideal candidate for gene therapy, which relies on the use of a gene transfer vector, typically viral, for the introduction of the corrected copy of the mutated gene. Several studies using different preclinical animal models and new data from recent clinical trials have demonstrated that these approaches are promising for the treatment of hemophilia patients (54). During the years, several efforts have been focused on viral vector designs in order to improve gene delivery and, at the same time, reduce or avoid immune reaction against the transgene (55). Strategies applied include targeted gene transfer, by transcriptional and post-transcriptional regulation, and shielding of the vector or transgene (55–58).

Several preclinical animal models of HA are presently available which can be used for the development of new gene therapy strategies to treat HA, to evaluate safety, as well as for dosage and long-term follow-up studies. These models include HA animals with spontaneous mutations, such as dogs, sheep and rats, and genetically engineered animals, like mice and pigs (59). Moreover, the generation of a HA mouse model carrying the human HLA class II antigen, associated in humans with higher inhibitor development risk, has given us the possibility to better understand/characterize mechanisms involved in immune reaction against FVIII (20, 60).

The liver is the major source of FVIII within the body (61, 62). Additionally, this organ is constantly balancing pro- and anti-inflammatory responses due to the continuous exposure to external antigens through the blood coming from the gut, thus creating a tolerant environment (63, 64). Several studies and results from clinical trials have demonstrated that liver-directed gene therapy for hemophilia is effective in correcting the HA bleeding phenotype (56, 65–69) taking advantage of the liver's tolerogenic ability (70–72). Moreover, targeted FVIII expression following gene therapy have been demonstrated to be successful even in presence of pre-existing inhibitors (69, 73, 74).

When considering gene delivery, recombinant adeno-associated viral (AAV) vectors have been used extensively in preclinical and clinical studies for FVIII expression in hepatocytes (65, 75, 76), as they are not integrating viral vectors, they can be produced with high yields and they are capable of long-term stable transgene expression in developed liver, while transgene expression results unstable and eventually lost in developing liver due to the non-integrating nature of AAV (77). Using AAV, Sabatino and colleagues have shown that liver-directed canine (c) FVIII gene therapy resulted in tolerance in HA dogs, with only 1 animal showing transitory development of low-titer inhibitors (2.5 BU) which was resolved at 7 weeks. This strategy resulted in detectable cFVIII activity and antigen levels as well as a reduction in whole blood clotting time (WBCT) in treated dogs. Further, tolerance to cFVIII in these HA dogs was maintained even following challenges with plasma-derived or recombinant cFVIII (68). The same group showed that the AAV liver-directed gene transfer is able to eradicate pre-existing high-titer inhibitors in HA dogs (69) and immune tolerance was still present in these animals several years after the first report (56).

Likewise, in HA mice, hepatocyte-directed FVIII gene therapy using AAV resulted in sustained therapeutic transgene expression avoiding inhibitor formation. The induction of tolerance was, however, directly correlated with the transgene expression levels, showing that high levels of FVIII expression are required in hepatocyte-directed gene therapy in order to avoid immune responses (78, 79). On the other hand, despite the initial high-level FVIII expression in hepatocyte-targeted FVIII gene therapy, a strong immune response is observed and inhibitors are developed following naked DNA transfer in HA mice (80). Moreover, it has been demonstrated that high levels of FVIII expression in hepatocytes are associated with transient endoplasmic reticulum stress and the consequent activation of unfolded protein response, with a correlation between FVIII expression and inhibitor formation (81, 82).

The liver is the main FVIII-producing organ and historically hepatocytes considered the principal site of FVIII synthesis. In more recent studies, however, it has been shown that the main FVIII-producing cells are the endothelial cells, particularly liver sinusoidal endothelial cells (LSEC) (83–85), and, to a lesser extent, hematopoietic cells (85, 86). Within the liver, LSEC were shown to be able to interact with T cells and modulate immune responses by preventing antigen-specific activation of CD8+ T cells, inhibiting the effector function of activate T cells and inducing Tregs (87, 88). Carambia and colleagues previously showed that LSECs are able to inhibit the pro-inflammatory activity of CD4 T cells through a IL-10- and PD-1-dependent mechanism (89). Moreover, these cells are able to retain TGF-β on their membrane and to induce antigen-specific CD4+CD25+Foxp3+ hepatic Tregs (88, 90). Our group recently demonstrated that targeting FVIII expression in endothelial cells, mainly LSEC, using a lentiviral vector (LV) containing the endothelial-specific vascular endothelial cadherin (VEC) promoter, results in sustained expression of therapeutic levels of FVIII in HA mice without inhibitor formation, even after immunization. This approach was demonstrated to be effective even in presence of and was able to revert pre-existing immunity to FVIII, suggesting a mechanism of Treg induction, since temporary depletion of Tregs resulted in a loss of FVIII activity and inhibitor formation. When Treg levels were returned to normal, inhibitor titers decreased and FVIII activity was restored to levels observed prior to Treg depletion (73). In the same study, targeting myeloid cells using the CD11b (integrin αM, ITGAM) promoter, long term FVIII expression was achieved but 30% of treated mice developed inhibitors, which were only avoided by de-targeting transgene expression in plasmacytoid dendritic cells using target sequence for microRNA-126 (73). In a more recent study, our group targeted FVIII expression in naturally FVIII-producing cells by generating a LV containing the transgene under the transcriptional control of the F8 promoter (pF8). Gene therapy in HA mice using this LV resulted in sustained production of therapeutic levels of FVIII. The levels of HA correction obtained with this strategy were higher when compared to those observed in our previous study targeting specifically endothelial cells with a typical endothelial promoter such as VEC. pF8 demonstrated to be active in an organ-dependent manner allowing transgene expression in hepatic endothelial cells as well as in splenic hematopoietic cells. Once again, with this strategy no inhibitors were observed and stable tolerance was reached via a mechanism involving Treg induction, even following FVIII challenges. Overall, this strategy was able to provide sustained FVIII therapeutic levels in HA mice with FVIII pre-existing immunity (74).

These data suggest that targeting FVIII transgene expression in cell types naturally producing FVIII represent a more effective strategy to avoid immune reaction and achieve tolerance to the transgene.

Direct intraosseous infusion of LV for the delivery of FVIII transgene in bone marrow (BM) and platelet-specific FVIII expression has been reported to be efficacious for the long-term treatment of the bleeding phenotype in hemophilia A mice. Using this strategy, Wang and colleagues designed a LV containing a FVIII transgene under the control of a platelet-specific promoter, the glycoprotein-1bα (GP1bα) promoter. Upon LV injection, no FVIII activity was detected in plasma of mice treated, while FVIII was present in ~2% of platelets by day 160 after LV delivery. On the contrary, after injection of the control vector, containing the FVIII sequence under the control of the ubiquitous human elongation factor 1α (EF-1α) promoter, they detected FVIII activity in circulation that subsequently decreased to undetectable levels due to inhibitors formation. Since FVIII is stored in α-granules (91), platelet-restricted FVIII expression shielded the presence of FVIII in circulation and resulted in long-term FVIII expression even in presence of high titer inhibitors (92).

An alternative approach to prevent immune responses to delivered transgene is delivering them during the neonatal period, allowing a tolerance induction to the transgene. Hu et al. showed sustained long-term FVIII expression (>5% for more than 1.5 year) following AAV-FVIII gene therapy in newborn (48 h/2 days old) HA mice. Tolerance to FVIII was reached with this strategy since immunization with FVIII in presence of an adjuvant at 8 weeks of life did not result in inhibitor formation. This study demonstrated the presence of the vector genome for more than 1 year, even though the vector copies drastically decreased after 8 weeks (>100-fold) and at the final time point, 1.5 years, were more than 400-fold lower (93). This is not surprising due to the non-integrating nature of AAV. For this reason, as an alternative strategy for the gene transfer in neonates avoiding vector genome “dilution” during the growth, the use of LV could be advantageous. In fact, LV has been shown to be effective in gene therapy approaches for other genetic diseases, such as Mucopolysaccharidosis type 1 (MPS 1) (94) and Pompe disease (95), without immune reactions against the transgene reported. Thus, LV-mediated FVIII gene therapy in HA neonates could represent a valid approach for the life-long treatment of the disease avoiding immune response and possibly inducing tolerance to FVIII.

An alternative and effective approach to obtain therapeutic levels of FVIII, while avoiding anti-FVIII immune responses, is represented by *ex vivo* gene therapy using hematopoietic stem cells (HSC). This strategy was shown to be able to provide a life-long transgene expression (96), in combination with transcriptional and post-transcriptional sequences to obtain lineage- or cell-type-specific transgene expression. HSC gene therapy is generally performed by transducing *ex vivo* HSC and transplanting them into conditioned recipients. Taking advantage of cell-type-specific transgene expression in HSC it is possible to obtain therapeutic FVIII expression avoiding immune reactions. For example, megakaryocyte-restricted FVIII expression using a lentiviral vector containing the integrin subunit αIIb (ITGA2B) promoter (2bF8) was able to ensure sustained long-term correction of the bleeding phenotype in HA mice (58, 97, 98) and dogs (99) without formation of anti-FVIII antibodies. This strategy was effective even in presence of inhibitors, since the synthesized FVIII was confined to the platelets, thus shielding the presence of FVIII in circulation and allowing its release following platelet activation to the injury site (58, 97, 98). Additional experiments following platelet-specific ovalbumin (OVA) expression (2bOVA) demonstrated that exists a natural peripheral tolerance to content of platelet granules, able to eliminate antigen-specific CD4 T effector cells and induce/expand antigen-specific Tregs (100), in agreement with a previous study by Chen et al. showing that transplantation of 2bF8-transduced HSC is able to induce immune tolerance to FVIII in HA mice through a CD4+ T cell-mediated mechanism (101).

Whether HSC-directed gene therapy is able to induce tolerance to FVIII is still under debate, as the immune suppressive drugs/treatments could be misleading with respects to the evaluation of the immune system responsiveness (56). However, previous studies showed that following HSC-based platelet-specific gene therapy antigen-specific immune tolerance was achieved in both hemophilia A and B mice and treated animals maintained the ability to respond to the unrelated immunogen ovalbumin (OVA) (102, 103). These studies demonstrate the possibility to treat inhibitor positive HA patients without the need of ITI for achieving hemostasis.

## Conclusions

During recent years, several approaches have been described to avoid inhibitor formation as well as to induce FVIII tolerance with the potential of improving both the rate of success and reduce the drawbacks of existing ITI protocols in clinic.

The use of immune modulatory drugs or molecules has been demonstrated to be helpful in suppressing immune reactions and in driving the immune system toward FVIII tolerance. This strategy, however, requires additional and more informative data regarding the long-term effects of these immune suppressive treatments on the immune system and the adverse effects in general associated to the use of immunosuppressive drugs. These effects may occur at the time of treatment or following the completion of treatment, such as infections, malignancy, bone marrow suppression and cytopenia (104).

Immunization of pregnant women is considered beneficial not only for protecting mothers from infections, but also offer protecting antibodies through the transplacental passage of immunoglobulins. The presence of maternal antibodies, however, could interfere with the immune response of the newborn to the vaccine (105). Further, the eventual presence of non-neutralizing anti-FVIII antibodies reported also in healthy individuals (106) could interfere with the administration of FVIII-Fc for transplacental FVIII delivery. Further studies aimed at deeply understanding and characterizing the mechanisms involved in transplacental transfer, will help in the development of molecules specifically designed for optimal delivery to the fetus and reduced interference from maternal antibodies.

Oral administration of antigens presents different advantages, including low costs, stability and self-administration, avoiding the discomfort of injectable preparations. This approach can be used for both immunization and tolerance induction according to the correct administration regimen (timing and dosage) (107). For these reasons, bioencapsulation of FVIII in plant cells and its oral administration would be an ideal alternative to current ITI protocols, giving the possibility of avoiding frequent injections (up to twice a day according to the protocol) and treatment-related high costs. One of the disadvantages of this strategy is that production of glycosylated antigens is not suitable in plastids (107), which are used to achieve high FVIII antigens production (42). Additional studies are necessary in order to better evaluate the efficacy of and the optimal dosage for this strategy in tolerance induction.

Among the described strategies is gene therapy, which despite the safety concerns related to genotoxicity and insertional mutagenesis, has the greatest potential. In fact, gene transfer could simultaneously prevent/eradicate inhibitors by tolerance induction and provide a life-long and sustained production of therapeutic FVIII levels with a single administration. This would reduce the high costs of existing substitution therapy and avoid the obligatory frequent FVIII infusions for HA patients. Between the vectors used in gene therapy, AAV have been used in numerous preclinical and clinical studies for hepatic FVIII expression (65, 75, 76). These vectors present some characteristics that make them attractive for gene delivery studies, including non-integrating ability, high yields during manufacturing processes and ability of long-term transgene expression (77). However, the use of AAV, especially in clinical trial, is limited by the presence of pre-existing immunity to AAV that could interfere with or nullify the gene transfer treatment and their non-integrating characteristic that is not optimal for the gene transfer in pediatric patients, which may not benefit from the therapy because of transgene dilution during the physiological liver growth. There are currently ongoing different phase 1–3 clinical studies for the treatment of hemophilia A using AAV (75). In 2017 Biomarin reported the first results of BMN270, a dose-escalation study conducted in nine patients, with stable FVIII after 1 year and very significant reduction in annualized bleed rate (from 16 to 1 event/year). They observed an elevation in alanine aminotransferase (ALT) levels in 8 out of 9 patients managed with corticosteroids with no effects on FVIII activity (65). More recently, Biomarin published an update of all the cohorts of this clinical trial reporting that most of the patients had substantial decrease in the occurrence of bleedings and more important the complete interruption of FVIII prophylaxis. These patients did not report liver damage even though liver-biopsy need to be taken in consideration to confirm the efficacy and safety of this approach (108).

Additional clinical trials from Spark (SPK-8011) and University College of London (GO-8) reported FVIII activity levels ranging from 13 to 30% for SPK-8011 and from 7 to 63% for GO-8. Both studies described an increase in ALT levels in some patients that were treated with corticosteroids. An high FVIII activity reduction was observed in two patients in the high-dose cohort of SPK-8011 following a capsid cellular immune response (75). Additional data from ongoing clinical studies after longer follow-up will help in clarify whether this approach, as previously observed in preclinical animal models, is able to induce tolerance to FVIII and allow a stable lifelong transgene expression, even though the corticosteroid treatment represent a confounder for the determination of immune tolerance induction.

Lentiviral vectors, on the other hand, are able to integrate within the host genome, have an expression cassette with doubled capacity compared to AAV, and present lower pre-existing immunity to LV elements (109, 110). These features allow the design of LV that can contain combinations of transcriptional and post-transcriptional regulation sequences, i.e., cell type-specific promoters and microRNA target sequences, in addition to the therapeutic transgene. This kind of approach allows transgene expression not only in determined cell types, but also in specific cell subpopulations, thus avoiding expression in unwanted cells and, in a final instance, immune reaction against the therapeutic transgene (55, 73, 74).

Several gene therapy studies are suggesting that antigen levels and a continuous transgene expression are involved in a successful tolerance induction to FVIII (56). Due to the high potential of this strategy of resolving HA and improving a patient's quality of life, future studies are necessary to improve and develop novel gene therapeutic tools.

While HA mice models have been fundamental for the design of the abovementioned strategies, additional preclinical studies in larger animal models are necessary to clarify the efficacy of these proposed approaches and to define the correct dosages and timing for their clinical use. Such models will also give the possibility of combining these different approaches and assessing their eventual long-term side effects.

Finally, several studies in recent years have highlighted the important involvement of the cells in the marginal zone of the spleen in early FVIII uptake and in the development of inhibitors in mice, including splenic follicular T cells, marginal zone B cells, marginal zone macrophages, and marginal zone metallophilic macrophages (26, 45, 111, 112). Despite this, the mechanisms of interaction between these cells in the induction of immune responses or tolerance to FVIII have yet to be described.

The understanding of cells involved in FVIII uptake and subsequent immune system activation as well as the mechanisms underlying the response to FVIII will contribute to refine the presented strategies, thus possibly reducing their eventual side effects, and will help the development of new therapies to prevent the formation of or revert existing inhibitors in HA patients.

## Author Contributions

AF and SM conceived the structure of the review, wrote the manuscript, and prepared the figure.

### Conflict of Interest

The authors declare that the research was conducted in the absence of any commercial or financial relationships that could be construed as a potential conflict of interest.
